# Measures of cellular oxidative damage following vitamin E supplementation in young patients with transfusion-dependent thalassemia: a double-blind randomized controlled trial

**DOI:** 10.1186/s12887-025-05741-2

**Published:** 2025-05-20

**Authors:** Nutthida Hemprachitchai, Rattanaporn Praneetponkang, Pakawan Wongwerawattanakoon, Chokdee Wongborisuth, Pawarit Innachai, Praguywan Kadegasem, Tanyanee Khlangtan, Thanaporn Sriwantana, Phanphen Phoonlapdacha, Oraporn Dumrongwongsiri, Ampaiwan Chuansumrit, Kovit Pattanapanyasat, Nongnuch Sirachainan, Nathawut Sibmooh, Pornthip Chaichompoo, Duantida Songdej

**Affiliations:** 1 Department of Pediatrics, Faculty of Medicine Ramathibodi Hospital, Mahidol University, 270 Rama VI Road, Ratchatewi District, Bangkok, 10400 Thailand; 2 Graduate Program in Molecular Medicine, Faculty of Science, Mahidol University, Bangkok, Thailand; 3 Department of Pathobiology, Faculty of Science, Mahidol University, Bangkok, Thailand; 4 Department of Nursing, Faculty of Medicine Ramathibodi Hospital, Mahidol University, Bangkok, Thailand; 5 Research Center, Faculty of Medicine Ramathibodi Hospital, Mahidol University, Bangkok, Thailand; 6 Chakri Naruebodindra Medical Institute, Faculty of Medicine Ramathibodi Hospital, Mahidol University, Samut Prakan, Thailand; 7 Center of Excellence for Microparticle and Exosome in Diseases, Department of Research and Development, Faculty of Medicine Siriraj Hospital, Mahidol University, Bangkok, Thailand

**Keywords:** Vitamin E, Oxidative stress, Red cell pathology, Thalassemia, PS-bearing RBC, Food supplements

## Abstract

**Background:**

Patients with thalassemia acquire cellular oxidative damage mainly from the degradation of excessive uncoupled hemoglobin (Hb) chains and iron overload. The oxidative damage of red blood cells (RBCs) and platelets potentially results in the worsening of ineffective erythropoiesis, hemolysis, and the occurrence of thromboembolic events. Vitamin E (VitE) is an antioxidant that inhibits membrane lipid peroxidation. It is widely used as a supplement in thalassemia; however, its benefits in improving cellular oxidative damage remain unclear.

**Methods:**

We conducted a double-blind, randomized, controlled trial registered in the Thai Clinical Trials Registry (TCTR20220801001) on 01/08/2022. We randomized transfusion-dependent (TD) β- and α-thalassemia (aged 10–25 years) to receive oral VitE 400 IU/day or placebo at a 1:1 ratio for 6 months. Cellular oxidative damage markers, including phosphatidyl serine (PS)-bearing RBCs, PS-bearing RBC vesicles, PS-bearing platelets, PS-bearing microparticles (MPs), PS-bearing RBC-MPs, PS-bearing platelet MPs (PMPs) and platelet activation, were measured before and after the intervention as the primary outcomes.

**Results:**

Seventy-four TD thalassemia patients were categorized into 63 β-thalassemia (10 splenectomy, β-Thal-S; and 53 non-splenectomy, β-Thal-NS) and 11 α-thalassemia (non-splenectomy, α-Thal-NS). Randomized from all patient groups, 36 received VitE and 38 received a placebo. A significant reduction in PS-bearing RBCs and PS-bearing RBC vesicles was observed in the β-Thal-NS receiving VitE. This occurred parallel with a substantial decrease in malondialdehyde levels, as a marker of lipid peroxidation, found only in the β-Thal-NS but not in β-Thal-S and α-Thal-NS groups. In the β-Thal-NS group, VitE had improved RBC pathology as demonstrated by the inverse correlation between post-treatment VitE levels and PS-bearing RBCs (*p* = *0.001)* as well as reticulocyte count (*p* = *0.006)*, although Hb levels remained unchanged. The VitE treatment did not result in improving platelet pathology or reducing MPs. No adverse event was reported in both VitE and placebo groups.

**Conclusions:**

VitE 400 IU/day was well-tolerated and associated with improved oxidative damage of the RBCs in TD β-Thal-NS patients. Accordingly, advice for VitE supplementation in young TD β-Thal-NS patients can be beneficial.

**Supplementary Information:**

The online version contains supplementary material available at 10.1186/s12887-025-05741-2.

## Introduction

Cellular oxidative damage occurs when the body has an imbalance of antioxidants and oxidants. This leads to the oxidation of cellular components such as lipids, proteins, and DNA, which causes cell damage [1]. The leading causes of cellular oxidative damage generated in thalassemia are the degradation of excessive, uncoupled and unstable hemoglobin (Hb) chains in erythroid precursors and iron overload. These subsequently aggravate the production of free radicals and lead to hemolysis [1–7]. Various forms of cellular oxidative damage occur in thalassemia patients, including blood cell membrane lipid peroxidation [2, 8] and generation of blood cell pathology, including phosphatidylserine (PS)-bearing cells [9], PS-bearing cell vesicles, formation of microparticles (MPs) (alternatively named membrane vesicles or medium extracellular vesicles) [10, 11] and platelet activation [12].

PS is a negatively charged phospholipid typically located in the inner layer of cell membranes. In thalassemia patients, excessive membrane lipid peroxidation of red blood cells (RBCs) and platelets results in a flip of PS from the inner to the outer layer of membranes. These pathologic cells are referred to as PS-bearing RBCs and PS-bearing platelets. In addition to the sequential worsening of ineffective erythropoiesis and hemolysis [13, 14], increased formation of PS-bearing RBCs, as well as PS-bearing platelets, were found to be associated with the hypercoagulable state in thalassemia patients by activating the coagulation cascade and stimulating platelets [9, 15]. MPs are small membrane vesicles, size 0.1–1 μm in diameter, shed from cells undergoing activation or apoptosis [11]. PS-bearing MPs stemming from PS-bearing RBCs and PS-bearing platelets were found at higher levels in β-thalassemia patients than in healthy individuals [16, 17]. Particularly, these PS-bearing MPs were significantly higher in splenectomized compared to non-splenectomized patients [16, 18]. Elevated level of MPs is an additional factor contributing to the hypercoagulable state and occurrence of thromboembolic events in β-thalassemia patients in reported literatures [15], as these MPs were shown to induce platelet activation, platelet aggregation, platelet-neutrophil aggregation and endothelial activation [10, 19].

Vitamin E (VitE) is an antioxidant that inhibits lipid peroxidation and reduces one of its by-products that serve as a biomarker of oxidative stress: malondialdehyde (MDA) [20]. Several reports found plasma VitE at lower levels in β-thalassemia patients than in healthy controls [12, 21, 22]. Many studies investigating the effects of VitE treatment on oxidative stress and antioxidant levels, as measured by various biomarkers, in transfusion-dependent (TD) and non-transfusion-dependent (NTD) β-thalassemia have been reported [21, 23–32]. Although most such studies showed improved oxidative status, the benefit of VitE treatment on improving hemolytic parameters and Hb levels was inconsistent. The potential benefits of VitE supplementation on ameliorating cellular oxidative damage in both β- and α-thalassemia patients remain lacking.

Herein, we conducted a double-blind, randomized, placebo-controlled trial with the primary aim of evaluating changes in cellular oxidative damage measures after 6-month supplementation of VitE in young patients with TD β- and α-thalassemia. We also explored changes in hemolytic parameters as secondary outcomes and nitrite levels as exploratory outcome measures.

## Methods

### Study design and participants

This double-blind, randomized, placebo-controlled clinical trial was conducted at the Department of Pediatrics, Faculty of Medicine Ramathibodi Hospital, Mahidol University, Bangkok, Thailand, from July 2022 to July 2023. The study protocol was approved by the local institutional review board (COA. MURA2022/150) and registered in the Thai Clinical Trials Registry (TCTR20220801001, first date of registration 01/08/2022). Patients with β- and α-thalassemia aged 10 to 25 years who required ≥ 7 RBC transfusions/year were enrolled. Those with VitE allergy or bleeding disorders and those requiring antioxidative agents as a part of essential medical treatments were excluded. Healthy controls were also recruited for baseline data comparison. Written informed consents were obtained from all participants and/or guardians of participants aged < 18. The study was conducted under the Declaration of Helsinki guidelines.

### Study protocol

**Fig. 1:**
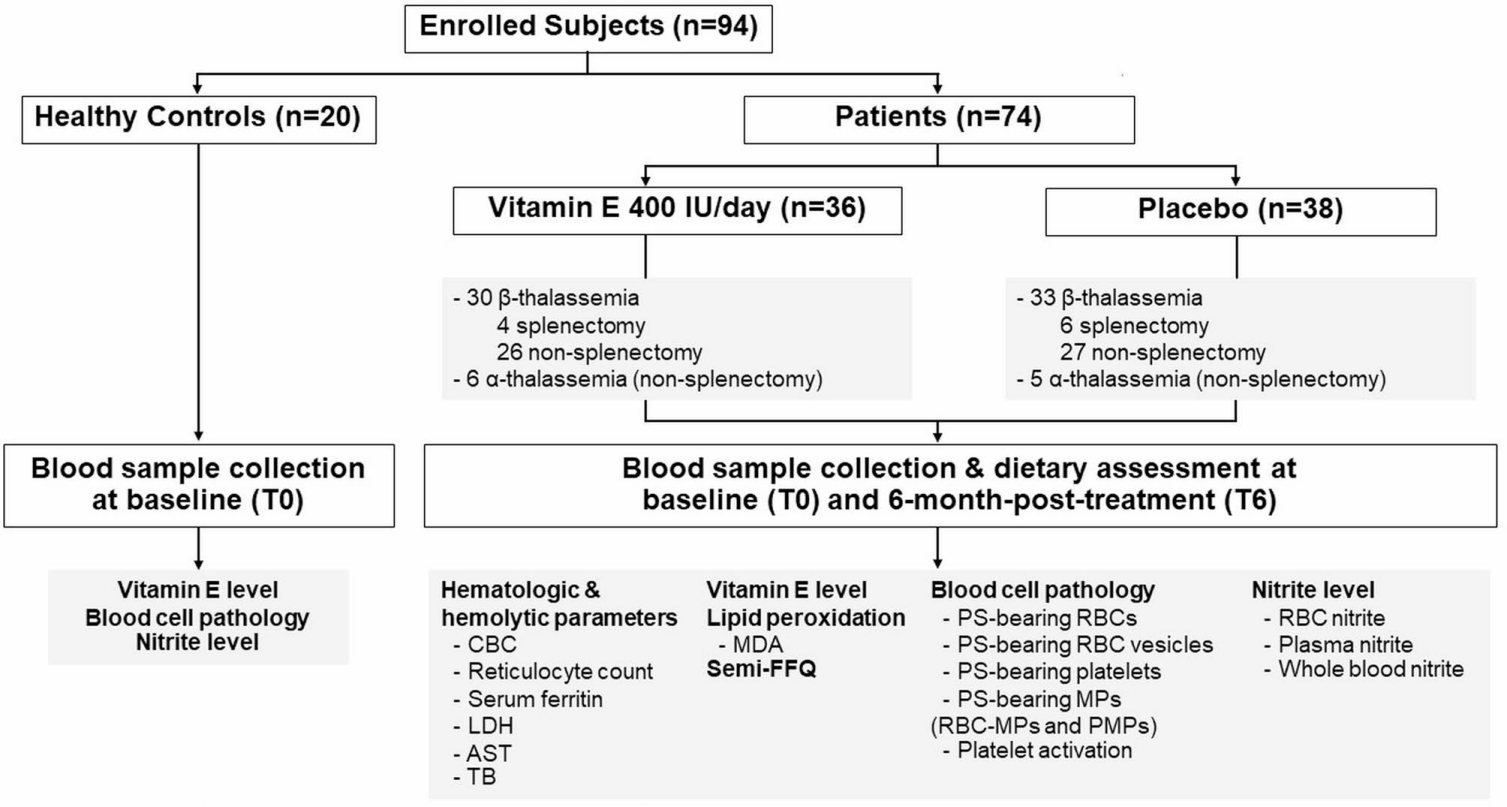
Diagram of the study workflow. Patients were randomized to receive vitamin E or placebo for 6 months. In addition to clinical data collection, blood samples were obtained for hematological and cellular oxidative damage analysis at baseline (T0) and post-treatment period (T6) in the patient group. Cellular oxidative damage analysis was also performed at a single timepoint (T0) in healthy controls for baseline comparison to the data derived from the patient group. Abbreviations: CBC, complete blood count; LDH, lactate dehydrogenase; AST, aspartate transferase; TB, total bilirubin; MDA, malondialdehyde; FFQ, food frequency questionnaire; PS, phosphatidyl serine; RBC, red blood cell; MPs, microparticles; PMPs, platelet microparticles

Enrolled patients were asked to stop medications or dietary supplement products considered direct antioxidative agents, such as VitE, Vitamin C (VitC), or N-acetylcysteine, if taken, for three months. As the baseline cellular oxidative damages could be significantly different between splenectomized and non-splenectomized patients [16, 18], these two groups of patients were subsequently randomized separately in a fixed block of four, using an online randomization tool to receive either oral VitE 400 IU/day or placebo (manufactured by Mega Lifesciences Ltd, Thailand) at 1:1 ratio for 6 months (Fig. 1). Compliance of VitE or placebo was assessed by records of the capsule return. All other standard treatments were continued during the trial. Patients were assessed at the hematology clinic every three months to evaluate adverse events by directly questioning the possible side effects of VitE.

### Clinical data and blood sample collection

Clinical and routine laboratory data were obtained from medical records. Additional blood samples were collected from the patients before the randomization (T0) and at the end of the intervention (T6). These samples were obtained in a fasting state and just before the transfusion. The samples were processed for measurements of VitE levels, MDA levels and cellular oxidative damage markers, including PS-bearing RBCs, PS-bearing RBC vesicles, PS-bearing platelets, PS-bearing MPs, PS-bearing RBC-MPs, PS-bearing platelet MPs (PMPs) and platelet activation. Moreover, levels of RBC, plasma and whole blood nitrite, a circulating nitric oxide (NO) metabolite, were analyzed to evaluate the possible association of VitE with the enhancement of NO bioavailability. In healthy controls, blood samples were collected at only one time-point (T0) and subjected to measurement of VitE levels, the cellular oxidative damage markers and nitrite levels.

All patients and/or their primary caregivers were asked to self-assess VitE and VitC dietary intakes using semi-food frequency questionnaires (FFQ) at T0 and T6.

### Laboratory assessment

Measurement of plasma VitE level was performed by high-performance liquid chromatography using HPLC ClinRep^®^ Complete Kits (RECIPE Chemicals + Instruments GmbH, Germany) as per manufacturer’s instruction. VitE deficiency was defined as VitE level < 5 mg/L. Serum MDA level was measured using a lipid peroxidation assay kit (Abcam, UK; Catalog number ab118970). Hemolytic parameters, including Hb level, reticulocyte count, lactate dehydrogenase (LDH), aspartate transaminase (AST), and total bilirubin (TB), were quantified using standard automated methods. The cellular oxidative damage markers were assessed using flow cytometric analysis (see Supplementary Methods). The nitrite levels were measured by tri-iodine-based chemiluminescence [33, 34] using the chemiluminescence NO analyzer.

### Statistical analysis

Data analysis was performed using IBM SPSS^®^ software, version 22. Descriptive data were presented as numbers (percentage) or mean (standard deviation, SD). A comparison of continuous variables was performed using the Student’s t-test or Mann-Whitney U test between two groups, as appropriate, and by ANOVA among more than two groups. A Spearman correlation coefficient was calculated to demonstrate the correlation between two continuous variables. A *p-value* less than 0.05 determined statistical significance.

## Results

### Characteristics of enrolled subjects and baseline cellular oxidative damages of thalassemic cells

A total of 74 patients (63 β- and 11 α-thalassemia) with mean (SD) age of 14.4 (2.7) years and 20 healthy controls were enrolled (Fig. 1). The patients were further categorized, according to their presence of spleen, into splenectomized β-thalassemia (β-Thal-S, *n* = 10), non-splenectomized β-thalassemia (β-Thal-NS, *n* = 53) and non-splenectomized α-thalassemia (α-Thal-NS, *n* = 11) groups for subsequent analysis. The baseline (T0) clinical and hematological characteristics of each patient group are shown in Table 1. In brief, patients had microcytic hypochromic hemolytic anemia and iron overload. All except three enrolled patients (α-Thal-NS) had been chelated, starting when their ferritin levels were ≥ 1,000 ng/mL. Deferiprone monotherapy is the first line iron chelator for patients aged ≥ 6 years in the country and was used in most patients in this cohort. Those who did not respond to deferiprone monotherapy and whose ferritin levels were ≥ 2,500 ng/mL were given deferasirox monotherapy or combination therapy of desferrioxamine/deferiprone or desferrioxamine/deferasirox or deferiprone/deferasirox as appropriate. Nevertheless, the mean ferritin levels remained ~ 2,000–3,000 ng/mL in patients of all diagnostic groups.

**Table 1:** Baseline clinical and laboratory characteristics of enrolled subjects

Characteristics	Healthy Controls (*n* = 20)	Patients			*P*-value
		β-Thal-S (*n* = 10)	β-Thal-NS (*n* = 53)	α-Thal-NS (*n* = 11)	
Age at enrollment (y)	14.4 (2.8)	15.7 (2.6)	14.2 (2.8)	14.4 (2.7)	*NS*
Sex: male (n, %)	9 (45)	4 (40)	25 (47.2)	5 (45.5)	*NS*
Diagnosis (n, %)					
β-thalassemia major	NR	1 (10)	4 (7.5)	NR	
β-thalassemia/HbE	NR	9 (90)	49 (92.5)	NR	
HbH-CS	NR	NR	NR	10 (90.9)	
EA Bart’s-CS	NR	NR	NR	1 (9.1)	
Age at initiation of regular transfusion (y)		1.8 (1.2)	3.0 (2.6)	4.2 (2.7)	*NS*
Frequency of transfusion (wk)	NR	3.4 (0.5)	3.6 (0.5)	4.2 (1.0)	*0.049*
Iron chelation (n, %)					
No chelator	NR	0	0	3 (27.3)	
Monotherapy	NR	7 (70)	42 (79.2)	8 (72.7)	
Combination therapy	NR	3 (30)	11 (20.8)	0	
Hemolytic parameters					
Hb (g/dL)	NA	9.8 (1.0)	9.3 (1.1)	8.9 (1.1)	*NS*
Reticulocyte count (%)	NA	12.6 (5.7)	2.7 (1.3)	7.8 (4.2)	*< 0.001*
LDH (U/L)	NA	154.7 (19.6)	248.6 (108.6)	376.9 (154.3)	*< 0.001*
AST (U/L)	NA	35.3 (17.6)	45.5 (37.3)	59.0 (19.6)	*0.022*
TB (mg/dL)	NA	2.6 (2.6)	3.0 (1.5)	2.8 (1.1)	*NS*
Ferritin level (ng/mL)	NA	3,065.4 (2,011.3)	2,967.8 (2,366.3)	2,253.0 (1,212.2)	*NS*
Vitamin E level (mg/L)	9.5 (1.4)	8.3 (1.6)	8.1 (2.4)	8.8 (3.4)	*NS*
MDA (nmol/mL)	NA	1.3 (0.9)	1.4 (0.9)	2.3 (0.5)	*0.021*

Data presented in mean (SD) unless specified otherwise

Abbreviations: β-Thal-S, splenectomized β-thalassemia; β-Thal-NS, non-splenectomized β-thalassemia; α-Thal-NS, non-splenectomized α-thalassemia; HbH-CS, hemoglobin H with hemoglobin Constant Spring (--/α^CS^α); EA Bart’s-CS, EA Bart’s with hemoglobin Constant Spring (--/α^CS^α, β^E^/β); Hb, hemoglobin; LDH, lactate dehydrogenase; AST, aspartate transaminase; MDA, malondialdehyde; y, year; wk, week; NR, not relevant; NA, not available; NS, not significant

**Fig. 2:**
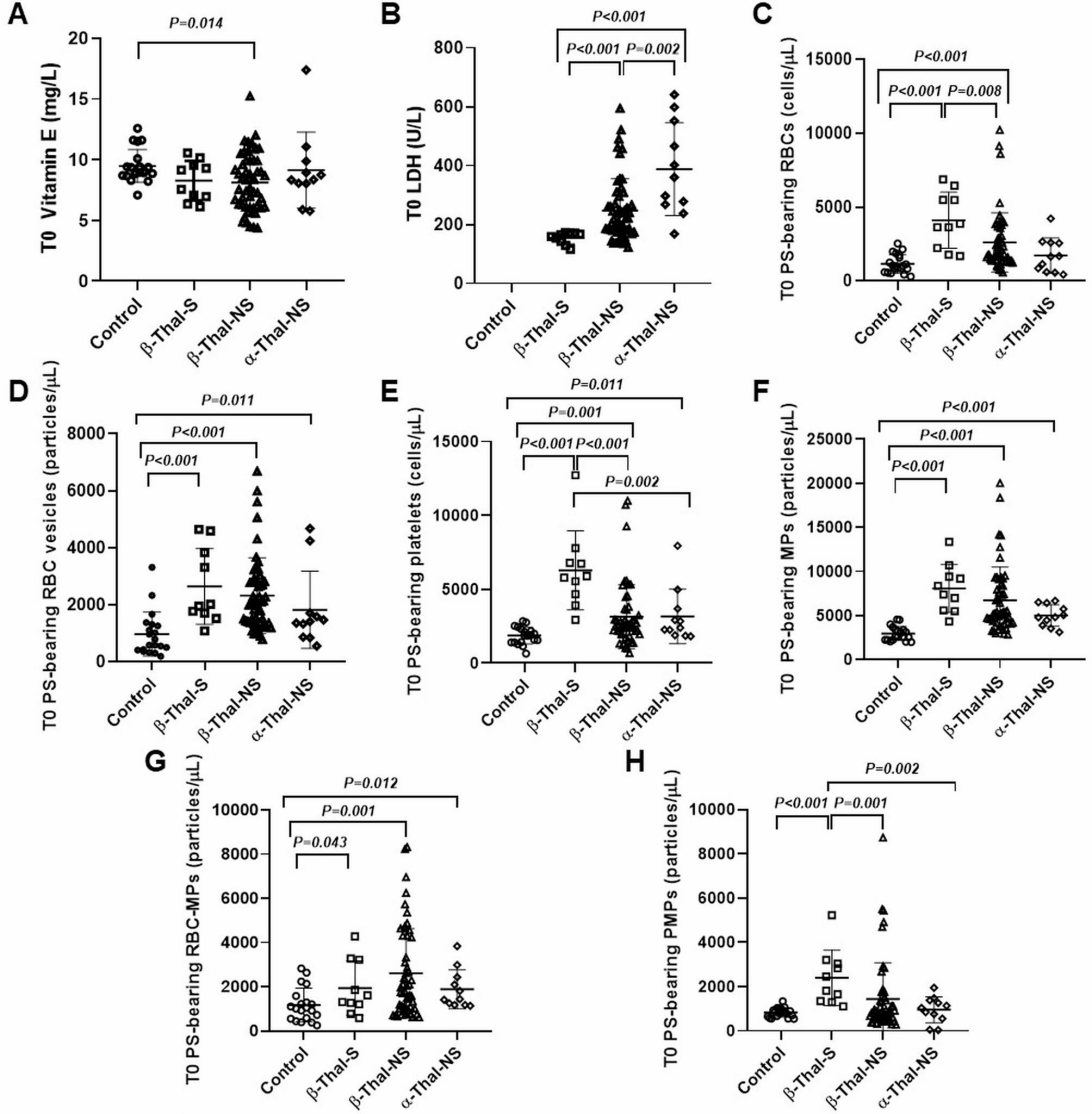
Comparison of baseline (T0) vitamin E level **(A)**, lactate dehydrogenase (LDH) **(B)**, Phosphatidyl serine (PS)-bearing red blood cells (RBCs) **(C)**, PS-bearing RBC vesicles **(D)**, PS-bearing platelets **(E)**, PS-bearing microparticles (MPs) **(F)**, PS-bearing RBC-MPs **(G)** and PS-bearing platelet MPs (PMPs) **(H)** among splenectomized β-thalassemia (β-Thal-S, *n* = 10), non-splenectomized β-thalassemia (β-Thal-NS, *n* = 53), non-splenectomized α-thalassemia (α-Thal-NS, *n* = 11) and healthy controls as indicated on the X-axis. Error bars represent the mean (SD) of each parameter

At T0, the mean (SD) VitE level of overall patients was significantly lower than that of the healthy controls [8.3 (2.4) vs. 9.5 (1.4) mg/L, *p* = 0.023]. This finding was remarkably observed in the β-Thal-NS group (Fig. 2A). However, none of the enrolled patients had VitE deficiency. Increased LDH was predominantly observed in β-Thal-NS and α-Thal-NS groups (Fig. 2B), corresponding with the ability of the intact spleen to remove thalassemic RBCs from the circulation; the process is known as extravascular hemolysis. Significantly higher numbers of PS-bearing RBCs, PS-bearing RBC vesicles, PS-bearing platelets, PS-bearing MPs and PS-bearing RBC-MPs were noted in the patient compared to the control groups (Fig. 2C-G). This suggested that oxidative stress generated in thalassemia patients generally resulted in variable damage to both RBCs and platelets. Additionally, patients in the β-Thal-S group were not only found to carry a significantly higher number of PS-bearing RBCs but also a higher number of PS-bearing platelets and PS-bearing PMPs compared to the other two patient groups (Fig. 2C, E, H). However, there was no significant difference in platelet activation observed in this study when comparing the β-Thal-S group and the other patient groups, as well as the controls (Supplementary Table S1).

### Characteristics of patients in VitE vs. placebo groups

In the intervention period, 4, 26 and 6 patients with β-Thal-S, β-Thal-NS and α-Thal-NS, respectively, were randomized to receive VitE, while the remaining patients were randomized to receive placebo (Fig. 1). Clinical characteristics of the patients in each diagnosis group, including age, sex, age at initiation of regular transfusion, frequency of transfusion and the use of iron chelators were comparable between those receiving VitE and placebo. Additionally, baseline (T0) pre-transfusion Hb level and the elevated serum ferritin were not significantly different between the two treatment groups as shown in Table 2. All enrolled patients completed a 6-month intervention period. Mean (SD) compliance of the investigational capsules was 81.6 (18.7)% in those receiving VitE, which was similar to 86.6 (15.8)% in those receiving placebo (*p* = *0.22)*. Among the patients receiving VitE, the mean (SD) daily doses were 9.6 (2.9), 10.6 (2.5) and 10.3 (2.3) IU/Kg body weight in β-Thal-S, β-Thal-NS and α-Thal-NS, respectively (*p* = *0.63*). No anticipated adverse effects of VitE or placebo, including nausea/vomiting, abdominal pain, diarrhea, headache, hypersensitivity reaction and abnormal bleeding, were reported during the study.

**Table 2:** Clinical characteristics of the patients in vitamin E and placebo groups

Characteristics	Vitamin E	Placebo	*P-value*
**β-Thal-S**	N = 4	N = 6	
Age at enrollment	17.0 (2.0)	14.8 (2.8)	*NS*
Sex: male (n, %)	2 (50)	2 (33.3)	*NS*
Diagnosis (n, %)			*NS*
β-thalassemia major	1 (25)	0	
β-thalassemia/HbE	3 (75)	6 (100)	
Age at initiation of regular transfusion (y)	1.8 (1.2)	1.8 (1.3)	*NS*
Frequency of transfusion (wk)	3.3 (0.5)	3.5 (0.5)	*NS*
Iron chelation (n, %)			*NS*
No chelator	0	0	
Monotherapy	2 (50)	5 (83.3)	
Combination therapy	2 (50)	1 (16.7)	
Hb (g/dL)	9.7 (0.8)	9.5 (1.4)	*NS*
Ferritin (ng/mL)	3,753.3 (2,081.3)	2,523.2 (1,850.7)	*NS*
**β-Thal-NS**	**N = 26**	**N = 27**	
Age at enrollment	13.5 (2.4)	14.8 (2.9)	*NS*
Sex: male (n, %)	10 (38.5)	15 (55.6)	*NS*
Diagnosis (n, %)			*NS*
β-thalassemia major	2 (7.7)	2 (7.4)	
β-thalassemia/HbE	24 (92.3)	25 (92.6)	
Age at initiation of regular transfusion (y)	2.4 (1.9)	3.5 (3.1)	*NS*
Frequency of transfusion (wk)	3.5 (0.5)	3.7 (1.4)	*NS*
Iron chelation (n, %)			*NS*
No chelator	0	0	
Monotherapy	20 (76.9)	22 (81.5)	
Combination therapy	6 (23.1)	5 (18.5)	
Hb (g/dL)	9.1 (1.1)	9.7 (1.0)	*NS*
Ferritin (ng/mL)	3,382.2 (2,572.7)	2,605.8.2 (2,173.7)	*NS*
**α-Thal-NS**	**N = 6**	**N = 5**	
Age at enrollment	14.0 (2.9)	14.9 (2.8)	*NS*
Sex: male (n, %)	3 (50)	2 (40)	*NS*
Diagnosis (n, %)			*NS*
HbH-CS	5 (83.3)	5 (100)	
EA Bart’s-CS	1 (16.7)	0	
Age at initiation of regular transfusion (y)	4.3 (3.3)	4.1 (2.3)	*NS*
Frequency of transfusion (wk)	4.2 (1.0)	4.2 (1.1)	*NS*
Iron chelation (n, %)			*NS*
No chelator	1 (16.7)	2 (40)	
Monotherapy	5 (83.3)	3 (60)	
Combination therapy	0	0	
Hb (g/dL)	9.4 (0.8)	8.3 (1.3)	*NS*
Ferritin (ng/mL)	2,157.5 (1,448.6)	2,372.3 (1,042.5)	*NS*

Data presented in mean (SD) unless specified otherwise

Abbreviations: β-Thal-S, splenectomize β-thalassemia; β-Thal-NS, non-splenectomized β-thalassemia; α-Thal-NS, non-splenectomized α-thalassemia; HbH-CS, hemoglobin H with hemoglobin Constant Spring (--/α^CS^α); EA Bart’s-CS, EA Bart’s with hemoglobin Constant Spring (--/α^CS^α, β^E^/β); Hb, hemoglobin; y, year; wk, week; NS, not significant

### Measures of cellular oxidative damage following VitE treatment

**Fig. 3:**
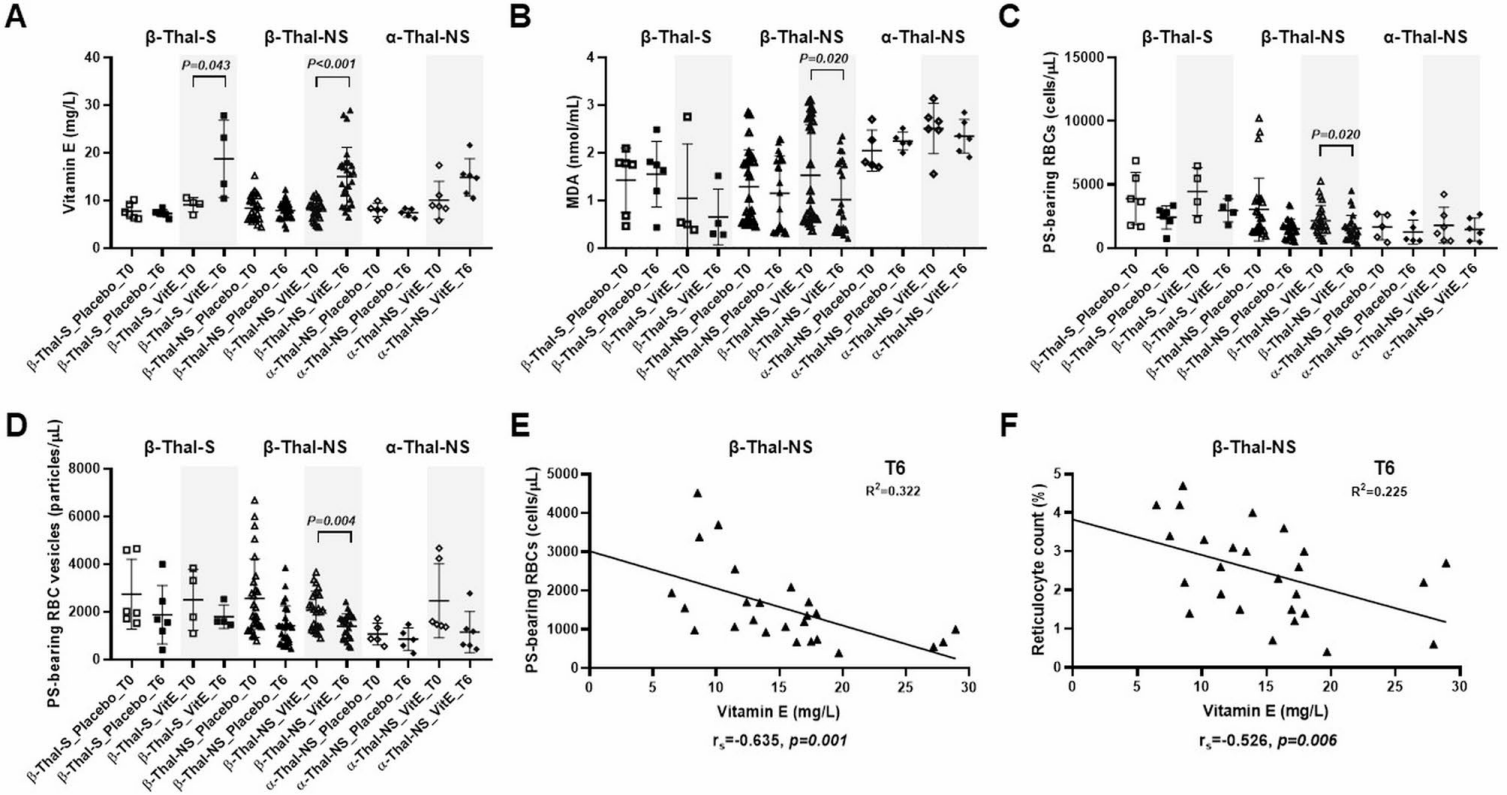
Scatter plots of baseline (T0) and post-treatment (T6) vitamin E level **(A)**, malondialdehyde (MDA) **(B)**, Phosphatidyl serine (PS)-bearing red blood cells (RBCs) **(C)** and PS-bearing RBC vesicles **(D)** in splenectomized β-thalassemia (β-Thal-S, *n* = 10), non-splenectomized β-thalassemia (β-Thal-NS, *n* = 53) and non-splenectomized α-thalassemia (α-Thal-NS, *n* = 11) patients as indicated on the X-axis. Error bars represent the mean (SD) of each parameter. Grey shades highlight patients randomized to the vitamin E treatment group. Correlation between post-treatment (T6) vitamin E level and PS-bearing RBCs **(E)**, as well as reticulocyte count **(F)** of β-Thal-NS patients are also demonstrated

VitE supplementation resulted in a significant increase in mean plasma VitE level at T6 compared to T0 in β-Thal-S and β-Thal-NS groups, while the mean VitE level of the patients receiving placebo in all diagnostic groups generally remained unchanged (Fig. 3A, Supplementary Table S2). However, a significant reduction in lipid peroxidation, as measured by MDA, was observed only in the β-Thal-NS group (Fig. 3B). Considering cellular oxidative damages, β-Thal-NS patients receiving VitE demonstrated significantly lower numbers of PS-bearing RBCs and PS-bearing RBC vesicles at post-treatment (Fig. 3C, D). Additionally, their plasma VitE levels at T6 inversely correlated with the number of PS-bearing RBCs (r_s_ = -0.635, *p* = 0.001) and the number of reticulocyte count (r_s_ = -0.526, *p* = 0.006) (Fig. 3E, F). Nevertheless, no significant change in Hb level was observed at the end of the study. In contrast, the numbers of PS-bearing platelets, PS-bearing MPs, PS-bearing RBC-MPs, PS-bearing PMPs and proportion of platelet activation in β-Thal-NS patients receiving VitE were not reduced and generally accumulated over time (Supplementary Table S2).

Supplementation of VitE 400 IU/day did not associate with reduction of cellular oxidative damages in β-Thal-S patients, who initially carried higher numbers of pathologic blood cells, including PS-bearing RBCs, PS-bearing platelets and PS-bearing PMPs, compared to the patients in β-Thal-NS and α-Thal-NS groups (Fig. 2, Supplementary Table S2). Moreover, the VitE treatment did not associate with a significant reduction of MDA or significant quantitative changes in the majority of measured cellular oxidative damages among α-Thal-NS patients. No improvement in Hb levels, as well as other hemolytic parameters, including reticulocyte count, LDH, AST, and TB, in β-Thal-S and α-Thal-NS patients receiving VitE were identified at the end of the intervention.

This study also demonstrated that RBC and whole blood nitrite levels significantly increased after 6 months of intervention in patients of all diagnostic groups. However, this finding did not occur only in patients receiving VitE but also in those receiving a placebo (Supplementary Table S2). This suggested a placebo effect on enhanced availability of the physiological storage form of NO. Dietary intakes of antioxidant vitamins, including VitE and VitC, were assessed at the two time points using semi-FFQ in patients of all diagnostic groups, and there was no difference between those receiving VitE and placebo (Supplementary Table S3). This implied that the dietary antioxidants were unlikely to contribute to the observed outcomes in this study.

## Discussion

This study primarily demonstrated that a 6-month supplementation of daily oral VitE 400 IU in young TD thalassemia patients was associated with improvement of cellular oxidative damages, specifically RBC pathology, including decreased numbers of PS-bearing RBCs and PS-bearing RBC vesicles. However, such an association was significantly observed only in the β-Thal-NS group. This occurred in parallel with a significant reduction in MDA levels found only in the β-Thal-NS but not in β-Thal-S and α-Thal-NS groups.

Most previous clinical studies suggested the benefit of VitE in generally reducing oxidative stress in β-thalassemia patients [21–23, 25, 27, 29, 31]. Other studies specifically showed a reduction of RBC MDA following 15 days to 16 months of either VitE alone [30, 31, 35] or a 4-month supplementation of VitE in combination with other antioxidative agents [24]. In alignment with these latter studies, we further demonstrated that oral VitE at 400 IU daily ameliorated RBC but not platelet pathology in the β-Thal-NS group. The significant inverse correlation identified between post-treatment VitE levels and the numbers of PS-bearing RBCs, as well as the proportion of reticulocyte count, suggested that this RBC pathology amelioration effects of VitE likely led to improvement of hemolysis in β-Thal-NS patients. These findings also suggested that reduction in lipid peroxidation following VitE treatment may occur in the RBC membrane as a primary site. However, pre-transfusion Hb levels were not shown to be increased in β-Thal-NS patients following the 6-month supplementation period. A previous study reported an increase in Hb level after 12 months of 400 IU/day VitE treatment [21], suggesting this clinically significant change might require a longer duration of intervention.

In β-Thal-S patients, the enhanced VitE level following supplementation tended to result in a reduction of MDA, a decrease of PS-bearing RBCs and PS-bearing RBC vesicles, although statistical significance was not observed. We hypothesized that a more remarkably observed initial amount of such RBC pathology in β-Thal-S patients, compared with patients of the other two diagnostic groups, might require a higher dose of VitE supplement to provide the noticeable improvement effects. This was supported by a small study showing reversal of impaired RBC osmotic fragility after 3- to 6-month VitE 750 IU/day supplementation in patients with splenectomized β-thalassemia major [36]. The current study additionally showed that patients in the α-Thal-NS group did not seem to benefit from the VitE treatment. It was possibly because α-Thal-NS patients primarily carried less cellular oxidative damage than β-Thal-S and β-Thal-NS patients. The exact reason behind this ineffectual outcome required further studies.

This study demonstrated that 400 IU/day VitE treatment could not decrease RBC and platelet-derived MPs in patients of all diagnostic groups. In contrast, these MPs accumulated throughout the disease. This was possible because the formation of MPs is influenced not only by oxidative stress but also by other factors such as inflammation, activation of coagulation or complement systems, and shear stress in the circulation [37]. We demonstrated that the VitE supplement did not associate with platelet activation/function changes in the β-Thal-S group. This was contrary to the finding in a previous report by *Unchern et al.* [12], in which the authors showed reduced platelet reactivity following 3-month VitE supplementation in splenectomized patients. This might be because their patients were supplemented with a higher dose of VitE (525 IU/day) and platelet reactivity in their study was measured by platelet aggregation test, while platelet activation in the current study was measured using flow cytometry. VitE was previously shown to have a protective effect in some ischemic injuries, such as ischemia-reperfused kidney injury, by enhancing the NO reservoir (nitrite) of ischemic tissue [38]. Nevertheless, it was found in this study that VitE did not provide a benefit over placebo treatment in elevating nitrite levels, hence preventing related ischemic effects in thalassemia patients.

The data at T0 revealed that young patients with both β- and α-thalassemia generally carried higher numbers of cellular oxidative damages of both RBC and platelet origins compared to controls, similar to that found in adult thalassemia patients [9, 16, 17]. This implied cellular pathology caused by oxidative stress accumulated since the patients were very young. However, patients in this cohort generally had high iron burden due to high transfusion to maintain Hb 9–10.5 g/dL in β- and 8–9 g/dL in α-thalassemia. This could have influenced the increase of oxidative damage of the blood cells observed at a young age in the study. A recent research supporting this showed an association between iron overload and oxidative stress on bone marrow erythropoiesis [7]. This data also implied that adequate iron chelation is of the utmost importance as a primary treatment to minimize oxidative damage. In agreement with related studies [9, 16, 18, 19], these pathologic RBCs and platelets were remarkably found in β-Thal-S patients, whose bodies could not remove such damaged thalassemic cells. The findings aligned with a recent study of similar populations, showing more severe oxidative stress and damage in splenectomized patients as measured by different markers [5]. The data showed that while α-Thal-NS patients had a more severe degree of hemolysis compared to those in the β-Thal-S and β-Thal-NS groups, as delineated by higher LDH, they generally suffered lower levels of cellular oxidative damage. This supported that the degradation of excessive unstable Hb chains and iron overload, the leading causes of cellular oxidative damage, occurred less in α-thalassemia, mainly non-deletional HbH, compared to β-thalassemia patients. Moreover, hemolysis in α-thalassemia patients might not only result from cellular oxidative damage but is more likely a multifactorial process.

In this study, we chose a single dose of VitE 400 IU/day during the intervention period across all enrolled patients since data on the safety of higher doses used in the long term among young patients were limited [31, 39]. While no patients suffered from VitE deficiency at baseline, this study showed that VitE supplement remained beneficial in reducing MDA and specific cellular oxidative damage measures, at least in the β-Thal-NS group. No adverse event of VitE was reported in this study, suggesting the 400 IU/day supplement was well-tolerated.

The strength of this study was the double-blind, randomized controlled trial study design, providing an unbiased assessment of both the outcomes and adverse effects and the inclusion of both TD β- and α-thalassemia. The limitations of this study were the use of only one dose of VitE in all patients, the relatively small number of patients and the short duration of intervention. Future large studies with higher doses of VitE given for at least > 12 months could be beneficial, especially for patients in the β-Thal-S group. Long-term studies are also required to determine whether the decreased numbers of PS-bearing RBCs and PS-bearing RBC vesicles in β-Thal-NS patients receiving VitE lead to a reduced risk of having thromboembolic events.

In conclusion, oral VitE (400 IU/day) supplementation for 6 months was well-tolerated and associated with a significant reduction in the numbers of PS-bearing RBCs and PS-bearing RBCs vesicles, which occurred in parallel with decreased MDA levels, in young TD β-Thal-NS patients. These resulted in an improvement in hemolysis, although the Hb level was unchanged. Therefore, VitE supplemented to young TD β-Thal-NS patients can be beneficial for reducing cellular oxidative damage, although more extensive studies with longer follow-up duration are required to emphasize the study results.

## Electronic supplementary material

Below is the link to the electronic supplementary material.

Supplementary Material 1

Supplementary Material 2

## Data Availability

All data generated or analyzed during this study are included in this article and its supplementary information files.

## Abbreviations

AST: Aspartate transaminase
FFQ: Food frequency questionnaires
Hb: Hemoglobin
LDH: Lactate dehydrogenase
MDA: Malondialdehyde
MPs: Microparticles
NO: Nitric oxide
NTD: Non-transfusion-dependent
PMPs: Platelet microparticles
PS: Phosphatidylserine
RBC: Red blood cell
SD: Standard deviation
TB: Total bilirubin
TD: Transfusion-dependent
VitC: Vitamin C
VitE: Vitamin E
α-Thal-NS: Non-splenectomized α-Thal
β-Thal-NS: Non-splenectomized β-Thal
β-Thal-S: Splenectomized β-Thal
