# Supplementary material for: Measures of cellular oxidative damage following vitamin E supplementation in young patients with transfusion-dependent thalassemia: a double-blind randomized controlled trial
Source: BMC Pediatr. 2025 May 20;25:405. doi: 10.1186/s12887-025-05741-2 (PMC12090501; doi:10.1186/s12887-025-05741-2)
Supplement: Supplementary file 1 — Supplementary Material 1 [file 12887_2025_5741_MOESM1_ESM.docx]

**Supplementary** **Methods**

***Preparation of vitamin E (VitE) and placebo capsules***

A placebo capsule was identical in size, shape and color to the VitE capsule without its active ingredient. VitE and placebo capsules were packed in sealed opaque bottles labeled with the trial number at a third-party location and transferred to the investigators for study use. Participants and all investigators were blinded regarding the treatment until the last patient completed the 6-month intervention period.

***Cellular* *oxidative damage profiles analysis***

PS-bearing cell and MP profiles, including their quantity and cellular origins, were determined by flow cytometric analysis. Briefly, diluted sodium citrate whole blood samples (dilution 1:10) were stained with FITC conjugated annexin V (PS marker; BD Biosciences), PE Cy5 conjugated anti-glycophorin A (RBC marker; BD Biosciences) and PE conjugated anti-CD41a (platelet marker; BD Biosciences). Particles with sizes comparable to 0.79-μm and 1.32-μm in diameter nano polystyrene beads (Spherotech) were used for the identification of MP populations. The absolute number of MPs (particles/μL) was calculated using TruCount beads (BD Biosciences) according to the formula below:

Absolute count = Number of positive cell events X Total bead per test X Dilution factor

Number of bead events Test volume

Data was acquired and analyzed using CellQuest Pro software, FACScan flow cytometer (BD Biosciences) as described in previous studies [1, 2].

***Platelet activation analysis***

Diluted whole blood samples were stained with FITC conjugated anti-CD41a and PE conjugated anti-CD62P as markers of platelets and activated platelets, respectively (BD Biosciences). The percentage of platelet activation was determined by CD41a^+^CD62P^+^ platelet portions. Data was acquired and analyzed using CellQuest Pro software, FACScan flow cytometer (BD Biosciences) as described in a previous study [3].

***Measurement of nitrite levels***

To explore whether VitE additionally has secondary effects on other oxidative stress-related physiologic pathways, specifically enhancement of nitric oxide (NO) production, in thalassemia patients, nitrite levels were measured. Nitrite is a circulating NO metabolite that can be converted to NO to facilitate vasodilation and tissue perfusion. In this study, nitrite levels in RBCs, plasma and whole blood were measured. Owing to an ultra-short half-life of nitrite (10 minutes), following blood collection at room temperature, the heparinized whole blood samples were mixed immediately with the nitrite-stabilizing solution containing 0.8 M ferricyanide, 10 mM N-ethylmaleimide and 1% NP-40 in a 4:1 ratio (v/v, sample/stabilizing solution) [4]. In a separate set of samples, the whole blood was centrifuged immediately at 14,000 x g for 2 minutes at 4^๐^C to obtain the packed RBCs and plasma. Plasma was separated and RBCs were immediately mixed with the nitrite-preserving solution prior to -80^º^C storage. The nitrite levels in the stored samples were subsequently measured in batches specifically by tri-iodine-based chemiluminescence [4, 5] using the chemiluminescence NO analyzer.

**Dietary assessment**

The dietary data obtained from self-assessment semi-food frequency questionnaires were calculated based on Thai food composition data and chemical analysis using INMUCAL-Nutrients Software, version 4.0, developed by the Institution of Nutrition, Mahidol University, Bangkok, Thailand. The adequacy of VitE and C intakes was assessed using the dietary reference intakes (DRIs) data for Thais 2020. VitE and VitC intake in the studied population were presented as a percentage of recommended amounts.

**References**

1. Manakeng K, Prasertphol P, Phongpao K, Chuncharunee S, Tanyong D, Worawichawong S et al. Elevated levels of platelet- and red cell-derived extracellular vesicles in transfusion-dependent β-thalassemia/HbE patients with pulmonary arterial hypertension. Ann Hematol. 2019;98(2):281-8.

2. Pattanapanyasat K, Gonwong S, Chaichompoo P, Noulsri E, Lerdwana S, Sukapirom K, et al. Activated platelet-derived microparticles in thalassaemia. Br J Haematol. 2007;136(3):462-71.

3. Keawvichit R, Khowawisetsut L, Chaichompoo P, Polsrila K, Sukklad S, Sukapirom K et al. Platelet activation and platelet-leukocyte interaction in β-thalassemia/hemoglobin E patients with marked nucleated erythrocytosis. Ann Hematol. 2012;91(11):1685-94.

4. Dejam A, Hunter CJ, Pelletier MM, Hsu LL, Machado RF, Shiva S, et al. Erythrocytes are the major intravascular storage sites of nitrite in human blood. Blood. 2005;106(2):734-9.

5. Feelisch M, Rassaf T, Mnaimneh S, Singh N, Bryan NS, Jourd'Heuil D, et al. Concomitant S-, N-, and heme-nitros(yl)ation in biological tissues and fluids: implications for the fate of NO in vivo. FASEB J. 2002;16(13):1775-85.
