# Supplementary material for: Measures of cellular oxidative damage following vitamin E supplementation in young patients with transfusion-dependent thalassemia: a double-blind randomized controlled trial
Source: BMC Pediatr. 2025 May 20;25:405. doi: 10.1186/s12887-025-05741-2 (PMC12090501; doi:10.1186/s12887-025-05741-2)
Supplement: Supplementary file 2 — Supplementary Material 2 [file 12887_2025_5741_MOESM2_ESM.docx]

**Supplementary Table S1.** Baseline cellular oxidative damages of enrolled subjects

| **Parameters** | **Healthy Controls**  **(n=20)** | **Patients** | | | ***P-value*** |
| --- | --- | --- | --- | --- | --- |
|  |  | **β-Thal-S**  **(n=10)** | **β-Thal-NS**  **(n=53)** | **α-Thal-NS**  **(n=11)** |  |
| Blood cell pathology |  |  |  |  |  |
| PS-bearing RBCs (cells/μL) | 1,165.4  (623.5) | 4,121.4 (1,891.0) | 2,613.1 (2,006.3) | 1,734.6 (1,183.2) | *<0.001* |
| PS-bearing RBC vesicles (particles/μL) | 970.5  (773.2) | 2,642.6 (1,324.8) | 2,322.9 (1,322.1) | 1,822.0 (1,350.9) | *<0.001* |
| PS-bearing platelets (cells/μL) | 1,869.6  (564.4) | 6,281.5 (2,677.2) | 3,152.7 (2,162.2) | 3,175.8 (1,842.0) | *<0.001* |
| PS-bearing MPs (particles/μL) | 2,970.9  (787.4) | 8,087.9 (2,708.7) | 6,750.5 (3,749.7) | 5,015.8 (1,221.5) | *<0.001* |
| PS-bearing RBC-MPs (particles/μL) | 1,177.6  (756.2) | 1,940.4 (1,229.3) | 2,609.3 (2,026.6) | 1,888.9 (879.7) | *0.010* |
| PS-bearing PMPs (particles/μL) | 833.3  (208.1) | 2,397.2 (1,255.9) | 1,439.6 (1,641.5) | 954.1  (588.5) | *0.002* |
| Platelet activation (%) | 3.1 (0.9) | 7.9 (15.8) | 2.9 (2.3) | 3.9 (2.1) | *NS* |
| Nitrite levels |  |  |  |  |  |
| RBC nitrite (nM) | 203.8 (109.2) | 168.7 (69.2) | 127.4 (37.2) | 148.6 (20.6) | *NS* |
| Plasma nitrite (nM) | 84.9 (46.8) | 81.1 (35.2) | 75.3 (43.6) | 81.4 (40.3) | *NS* |
| Whole blood nitrite (nM) | 125.9 (39.5) | 136.3 (51.2) | 143.6 (83.7) | 161.8 (49.0) | *NS* |

Data presented in mean (SD) unless specified otherwise

Abbreviations: RBC, Red blood cell; PS, phosphatidyl serine; MP, microparticle; PMP, platelet microparticle; NS, not significant

**Supplementary Table S2.** Changes of cellular oxidative damages in thalassemia patients from baseline (T0) to the end of study (T6)

| **Parameters** | **β-Thal-S (n=10)** | | **β-Thal-NS (n=53)** | | **α-Thal-NS (n=11)** | |
| --- | --- | --- | --- | --- | --- | --- |
|  | **Vitamin E (n=4)** | **Placebo (n=6)** | **Vitamin E (n=26)** | **Placebo (n=27)** | **Vitamin E (n=6)** | **Placebo (n=5)** |
| Hb (g/dL) |  |  |  |  |  |  |
| T0 | 9.7 (0.8) | 9.5 (1.4) | 9.1 (1.1) | 9.7 (1.0) | 9.4 (0.8) | 8.3 (1.3) |
| T6 | 10.0 (2.2) | 9.8 (1.0) | 9.0 (0.9) | 9.4 (1.1) | 9.0 (0.7) | 8.6 (1.0) |
| *P-value* | *NS* | *NS* | *NS* | *NS* | *NS* | *NS* |
| Reticulocyte count (%) |  |  |  |  |  |  |
| T0 | 11.3 (3.8) | 11.6 (8.1) | 2.7 (1.5) | 2.9 (1.1) | 7.7 (3.6) | 8.0 (5.6) |
| T6 | 9.8 (6.7) | 13.3 (6.2) | 2.5 (1.2) | 2.8 (1.4) | 7.7 (2.4) | 9.4 (2.8) |
| *P-value* | *NS* | *NS* | *NS* | *NS* | *NS* | *NS* |
| Ferritin level (ng/mL) |  |  |  |  |  |  |
| T0 | 3,753.3 (2,081.3) | 2,523.2 (1,850.7) | 3,382.2 (2,572.7) | 2,605.8 (2,173.7) | 2,157.5 (1,448.6) | 2,372.3 (1,042.5) |
| T6 | 4,195.4 (2,130.5) | 1,996.4 (1,309.0) | 3,493.3 (2,694.9) | 2,511.1 (2,072.4) | 1,875.2 (1,284.9) | 3,490.6 (3,983.5) |
| *P-value* | *NS* | *NS* | *NS* | *NS* | *NS* | *NS* |
| Vitamin E level (mg/L) |  |  |  |  |  |  |
| T0 | 9.1 (1.5) | 7.4 (1.7) | 8.0 (2.1) | 8.3 (2.6) | 9.8 (4.4) | 7.6 (1.1) |
| T6 | 18.7 (8.1) | 7.3 (0.8) | 15.3 (6.1) | 7.8 (1.8) | 14.8 (3.9) | 7.4 (0.9) |
| *P-value* | *0.043* | *NS* | *<0.001* | *NS* | *NS* | *NS* |
| MDA (nmol/mL) |  |  |  |  |  |  |
| T0 | 1.0 (1.1) | 1.3 (0.7) | 1.5 (1.1) | 1.3 (0.8) | 2.5 (0.6) | 2.1 (0.4) |
| T6 | 0.7 (0.6) | 1.6 (0.7) | 1.0 (0.7) | 1.2 (0.8) | 2.3 (0.4) | 2.2 (0.2) |
| *P-value* | *NS* | *NS* | *0.020* | *NS* | *NS* | *NS* |
| LDH (U/L) |  |  |  |  |  |  |
| T0 | 149.5 (26.1) | 160.4 (15.6) | 249.9 (128.5) | 250.2 (89.3) | 334.2 (96.2) | 430.3 (210.5) |
| T6 | 152.5 (40.1) | 151.3 (29.2) | 240.4 (118.8) | 223.7 (74.2) | 363.7 (112.5) | 381.8 (189.0) |
| *P-value* | *NS* | *NS* | *NS* | *NS* | *NS* | *NS* |
| AST (U/L) |  |  |  |  |  |  |
| T0 | 42.3 (25.6) | 36.9 (18.7) | 45.0 (46.9) | 44.9 (26.3) | 52.4 (16.4) | 67.2 (22.4) |
| T6 | 37.8 (22.4) | 27.1 (8.5) | 38.1 (18.6) | 38.5 (21.8) | 51.8 (19.5) | 94.4 (59.2) |
| *P-value* | *NS* | *NS* | *NS* | *NS* | *NS* | *NS* |
| TB (mg/dL) |  |  |  |  |  |  |
| T0 | 1.8 (0.7) | 2.9 (3.1) | 3.3 (1.7) | 2.8 (1.3) | 2.3 (1.0) | 3.5 (0.7) |
| T6 | 1.8 (1.1) | 3.2 (3.3) | 3.2 (1.5) | 2.3 (1.1) | 2.3 (1.8) | 2.8 (0.5) |
| *P-value* | *NS* | *NS* | *NS* | *NS* | *NS* | *NS* |

Data presented in mean (SD) unless specified otherwise

Abbreviations: β-Thal-S, splenectomize β-thalassemia; β-Thal-NS, non-splenectomized β-thalassemia; α-Thal-NS, non-splenectomized α-thalassemia; Hb, hemoglobin; MDA, malondialdehyde; LDH, lactate dehydrogenase; RBC, Red blood cell; PS, phosphatidyl serine; MP, microparticle; PMP, platelet microparticle; NS, not significant

**Supplementary Table S2.** (continued)

| **Parameters** | **β-Thal-S (n=10)** | | **β-Thal-NS (n=53)** | | **α-Thal-NS (n=11)** | |
| --- | --- | --- | --- | --- | --- | --- |
|  | **Vitamin E (n=4)** | **Placebo (n=6)** | **Vitamin E (n=26)** | **Placebo (n=27)** | **Vitamin E (n=6)** | **Placebo (n=5)** |
| Blood cell pathology |  |  |  |  |  |  |
| PS-bearing RBCs (cells/μL) |  |  |  |  |  |  |
| T0 | 4,454.5 (1,880.0) | 3,574.1 (2,052.7) | 2,151.7 (1,201.2) | 3,094.9 (2,516.5) | 2,044.5 (1,422.8) | 1,402.9 (954.7) |
| T6 | 2,953.7 (897.6) | 2,412.9 (917.3) | 1,554.0 (1,026.9) | 1,500.9 (765.0) | 1,460.3 (907.0) | 1,257.4 (951.0) |
| *P-value* | *NS* | *NS* | *0.020* | *0.002* | *NS* | *NS* |
| PS-bearing RBC vesicles (particles/μL) |  |  |  |  |  |  |
| T0 | 2,500.1 (1,282.6) | 2,753.4 (1,337.7) | 2,062.8 (799.2) | 2,552.8 (1,678.9) | 2,675.8 (1,632.1) | 1,187.1 (414.4) |
| T6 | 1,790.0 (496.3) | 1,872.7 (1,230.9) | 1,390.6 (516.0) | 1,411.3 (832.9) | 1,143.9 (865.4) | 849.6 (465.1) |
| *P-value* | *NS* | *NS* | *0.004* | *0.002* | *0.025* | *NS* |
| PS-bearing platelets (cells/μL) |  |  |  |  |  |  |
| T0 | 5,337.5 (1,656.9) | 6,202.2 (3,450.1) | 2,637.0 (1,196.7) | 3,694.8 (2,744.6) | 4,079.5 (2,434.0) | 2,464.8 (856.6) |
| T6 | 6,173.9 (2,554.5) | 6,227.2 (2,158.7) | 2,663.5 (823.6) | 2,997.9 (1,216.8) | 2,936.2 (1,893.6) | 2,173.1 (1,461.3) |
| *P-value* | *NS* | *NS* | *NS* | *NS* | *NS* | *NS* |
| PS-bearing MPs (particles/μL) |  |  |  |  |  |  |
| T0 | 7,358.0 (2,609.7) | 8,101.8 (2,928.0) | 5,276.2 (2,037.6) | 8,225.2 (4,491.5) | 4,499.0 (1,318.6) | 5,524.9 (1,292.9) |
| T6 | 14,186.6 (6,972.6) | 6,529.1 (2,865.5) | 6,921.8 (3,014.6) | 8,138.1 (3,000.4) | 6,847.2 (3,248.1) | 7,338.8 (6,362.0) |
| *P-value* | *NS* | *NS* | *0.021* | *NS* | *NS* | *NS* |
| PS-bearing RBC-MPs (particles/μL) |  |  |  |  |  |  |
| T0 | 1,611.1 (1,159.5) | 2,226.2 (1,226.6) | 1,826.5 (1,145.7) | 3,361.4 (2,432.5) | 1,804.1 (483.2) | 2,283.6 (1,346.8) |
| T6 | 4,115.3 (2,393.4) | 1,970.4 (1,545.6) | 2,555.5 (1,568.8) | 3,193.8 (1,927.8) | 3,286.3 (1,984.4) | 2,933.8 (2,999.0) |
| *P-value* | *NS* | *NS* | *NS* | *NS* | *NS* | *NS* |
| PS-bearing PMPs (particles/μL) |  |  |  |  |  |  |
| T0 | 2,105.0 (920.4) | 2,310.5 (1,550.0) | 896.4 (539.3) | 1,993.3 (2,139.5) | 686.9 (785.7) | 1,290.5 (192.4) |
| T6 | 5,909.4 (2,212.3) | 2,608.9 (1,278.2) | 2,175.8 (1,253.9) | 2,596.5 (1,227.4) | 3,512.1 (2,594.7) | 3,490.8 (3,926.7) |
| *P-value* | *0.043* | *NS* | *<0.001* | *0.006* | *0.010* | *NS* |
| Platelet activation (%) |  |  |  |  |  |  |
| T0 | 15.0 (25.1) | 2.8 (2.1) | 3.0 (2.0) | 2.9 (2.6) | 2.9 (1.6) | 5.1 (2.5) |
| T6 | 4.2 (1.2) | 3.4 (1.2) | 3.6 (2.0) | 3.2 (1.9) | 1.7 (0.8) | 3.6 (2.7) |
| *P-value* | *NS* | *NS* | *NS* | *NS* | *NS* | *NS* |
| Nitrite levels |  |  |  |  |  |  |
| RBC nitrite (nM) |  |  |  |  |  |  |
| T0 | 162.3 (79.2) | 171.6 (63.3) | 122.3 (31.5) | 131.0 (42.2) | 158.0 (23.6) | 136.9 (7.4) |
| T6 | 248.5 (39.5) | 260.7 (45.7) | 262.6 (64.7) | 258.4 (72.7) | 305.0 (98.0) | 250.7 (59.2) |
| *P-value* | *NS* | *0.046* | *<0.001* | *<0.001* | *0.004* | *0.009* |
| Plasma nitrite (nM) |  |  |  |  |  |  |
| T0 | 82.2 (54.8) | 83.5 (20.5) | 82.4 (53.7) | 67.4 (30.9) | 61.4 (35.0) | 106.4 (34.5) |
| T6 | 82.2 (41.2) | 90.4 (53.4) | 87.3 (37.8) | 94.4 (60.9) | 62.2 (28.1) | 69.0 (35.2) |
| *P-value* | *NS* | *NS* | *NS* | *NS* | *NS* | *NS* |
| Whole blood nitrite (nM) |  |  |  |  |  |  |
| T0 | 159.4 (50.8) | 137.8 (63.6) | 143.3 (90.4) | 140.2 (77.9) | 145.8 (42.2) | 181.9 (55.3) |
| T6 | 247.9 (18.4) | 276.8 (79.9) | 239.1 (68) | 276.8 (89.3) | 316.2 (125.9) | 254.5 (75.5) |
| *P-value* | *0.021* | *0.010* | *<0.001* | *<0.001* | *0.006* | *NS* |

**Supplementary Table S3.** Dietary vitamin E and C intakes among patients in vitamin E and placebo group

| **Parameters** | **β-Thal-S (n=10)** | | **β-Thal-NS (n=53)** | | **α-Thal-NS (n=11)** | |
| --- | --- | --- | --- | --- | --- | --- |
|  | **Vitamin E (n=4)** | **Placebo (n=6)** | **Vitamin E (n=26)** | **Placebo (n=27)** | **Vitamin E (n=6)** | **Placebo (n=5)** |
| Vitamin E (%DRI) |  |  |  |  |  |  |
| T0 | 5.5 (4.4) | 4.0 (1.1) | 3.4 (4.6) | 2.5 (3.2) | 6.6 (5.2) | 9.3 (9.7) |
| T6 | 2.9 (1.7) | 4.6 (2.2) | 4.3 (3.8) | 3.7 (4.0) | 3.1 (2.4) | 15.6 (13.6) |
| *P-value* | *NS* | *NS* | *NS* | *NS* | *NS* | *NS* |
| Vitamin C (%DRI) |  |  |  |  |  |  |
| T0 | 97.1 (48.1) | 191.2 (104.2) | 194.9 (184.7) | 99.7 (121.1) | 347.9 (216.4) | 253.0 (163.3) |
| T6 | 137.7 (76.9) | 231.7 (210.3) | 187.5 (145.5) | 113.1 (113.8) | 279.2 (146.5) | 288.0 (207.6) |
| *P-value* | *NS* | *NS* | *NS* | *NS* | *NS* | *NS* |

Data presented in mean (SD) unless specified otherwise

Abbreviations: DRI, Thai Dietary Reference Intakes; NS, not significant
